# Comparative Analysis of Whole-Body Diffusion-Weighted Imaging and PET/CT in Metastasis Detection: A Prospective Study

**DOI:** 10.7759/cureus.74756

**Published:** 2024-11-29

**Authors:** Betul Akdal Dolek, Deniz Sozmen Ciliz, Nuriye Ozdemir, Gulsum Ozet, Semra Duran

**Affiliations:** 1 Radiology, Ankara Bilkent City Hospital, Ankara, TUR; 2 Oncology, Gazi University, Ankara, TUR; 3 Hematology, Ankara Bilkent City Hospital, Ankara, TUR

**Keywords:** cancer staging, imaging technics, metastasis detection, pet/ct, whole body diffusion weighted mri

## Abstract

Introduction

Tumor staging is essential for determining treatment strategies and predicting prognosis in cancer patients. Accurate imaging techniques are critical for staging, metastasis screening, treatment response assessment, and recurrence detection.

Objective

In this prospective study, we aimed to compare the sensitivity of whole-body diffusion-weighted imaging (WB-DWI) with positron emission tomography/computed tomography (PET/CT) in detecting metastases.

Materials and methods

Twenty-one patients with metastatic cancer disease confirmed by PET/CT examination were prospectively examined with WB-DWI. PET/CT scans were performed using 18-fluorodeoxyglucose (18F-FDG). To assess and localize metastatic lesions, each case was evaluated at 18 different sites, including the skeletal system, visceral areas, and lymph nodes. Lesions were localized and counted using both radiological modalities.

Results

Twenty-one patients with metastatic disease were included (12 men, 9 women; mean age 58 years). All patients underwent PET/CT followed by WB-DWI. The average interval between PET/CT and WB-DWI was 22.5 days. Of the 378 regions examined, PET/CT detected 68 metastases, while WB-DWI detected 64 metastases. Liver metastases were detected in 2 out of 3 patients by WB-DWI. WB-DWI demonstrated substantial agreement with PET/CT in detecting liver metastases (κ (Cohen's kappa) = 0.77, p < .001) and lung metastases (κ = 0.74, p < .001). All adrenal gland and soft tissue metastases were detected by WB-DWI, with perfect agreement (κ = 1, p < .001). WB-DWI detected 31 metastatic lymph nodes identified by PET/CT, also with perfect agreement (κ = 1, p < .001).

Conclusion

WB-DWI offers significant advantages over PET/CT, including reduced imaging time, no radiation exposure, and lower costs. WB-DWI demonstrates comparable sensitivity to PET/CT in metastasis screening, suggesting the potential to reduce PET/CT usage with further improvements in DWI parameters and MRI technology.

## Introduction

Accurate tumor staging is pivotal for determining optimal treatment strategies and predicting prognosis in cancer patients. Imaging modalities are indispensable in this process, facilitating the detection of metastatic disease, evaluation of treatment response, and identification of disease recurrence.

Positron emission tomography/computed tomography (PET/CT) is widely regarded as the benchmark for detecting and characterizing metastatic disease. It is highly valued for its sensitivity and specificity in oncology, playing a crucial role in tumor staging and follow-up. However, PET/CT is not without limitations, including exposure to ionizing radiation, the need for on-site radionuclide production facilities, and challenges arising from physiological variability in fluorodeoxyglucose (FDG) uptake. Furthermore, respiratory motion artifacts and suboptimal accuracy in specific anatomical regions, such as the brain and urinary system, may reduce its diagnostic reliability.

In contrast, MRI offers high-resolution imaging without the risks associated with radiation exposure, making it an increasingly attractive option for oncological imaging. Recent advances in whole-body MRI (WB-MRI) technology, particularly the integration of diffusion-weighted imaging (DWI), have improved its utility for detecting primary tumors and metastatic lesions and monitoring treatment response. DWI uses variations in the diffusion of water molecules to identify changes associated with malignancy, such as increased cell density and altered extracellular space. The WB-DWI technique has enabled a novel approach to analyzing tumors affecting the entire body [1,2].

This prospective study aims to assess the diagnostic performance of WB-DWI for detecting metastatic lesions, using PET/CT as the reference standard modality. Metastases were confirmed by follow-up imaging and histopathological analysis where available, providing a robust evaluation of WB-DWI sensitivity. This study also explores the potential of WB-DWI to complement or even serve as an alternative to PET/CT in oncological imaging.

## Materials and methods

This study protocol was reviewed and approved by the Ankara Numune Training and Research Hospital Scientific Research Evaluation Board (approval number B.10.4.ISM.4.06.00.13).

Patients with metastatic cancer who had undergone or were planned to undergo PET/CT examination were included in the study. A total of 21 patients with metastatic disease were enrolled. All patients underwent both PET/CT and WB-DWI, with the average time between the two examinations calculated as 22.5 days. During the interval between the PET/CT and WB-DWI with background body signal suppression (WB-DWIBS) scans, none of the patients received any specific treatment (chemotherapy, radiotherapy, or surgery) for their cancer. Metastatic lesions were confirmed through follow-up imaging, and in cases of suspected malignancy, histopathological examination was performed to ensure diagnostic accuracy.

This study was conducted prospectively, and informed consent was obtained from all patients prior to the procedures.

PET/CT protocol

Patients underwent whole-body PET/CT imaging after adhering to a standardized preparation protocol. To ensure optimal tracer distribution and minimize variability, all patients fasted for at least six hours prior to the imaging session. Serum glucose levels were measured at the time of imaging to confirm they were below 150 mg/dL. Each patient received an intravenous injection of 18F-fluorodeoxyglucose (18F-FDG) at a standardized dose of 144 μCi/kg body weight. Whole-body PET/CT imaging was initiated 60 minutes post-injection to allow for adequate tracer uptake.

CT scans were performed without contrast enhancement using a multi-detector PET/CT scanner (Biograph 6; Siemens Medical Systems, Erlangen, Germany). Acquisition parameters for CT imaging included a tube voltage of 130 kV, a mean tube current of 90 mA, and a section thickness of 3 mm. These CT images served dual purposes: anatomical localization and attenuation correction for PET images.

PET emission scans were acquired immediately following CT imaging. The scan covered the whole body in 5-7 bed positions, with an acquisition time of four minutes per bed position.

The PET images were reconstructed using an iterative reconstruction algorithm with CT-based attenuation correction to enhance image quality and resolution.

Two experienced nuclear medicine physicians performed both visual and quantitative evaluations of the PET/CT images. Metastatic lesions were identified, localized, and quantified on these images, with their maximum standardized uptake values (SUVmax) measured. Lesions were defined as distinct structures exhibiting an SUVmax greater than 2.5.

MRI

The WB-DWI examinations were performed using a 1.5T MR scanner (Optima, GE HealthCare, Waukesha, Wisconsin). Following the WB-DWI technique, all patients underwent whole-body short inversion time inversion recovery (WB-STIR) imaging to assist with the anatomical localization of lesions.

Intravenous contrast material was not used during the examination. Anesthesia was not required for any patient, and all participants tolerated the examination well.

Similar to PET/CT, images were obtained from the head vertex to the mid-thigh. The examination area was divided into four stations: head-neck, chest-upper abdomen, lower abdomen, and proximal thigh. At the conclusion of the WB-DWI, the arms of all patients were positioned in the anatomical position, and a WB-STIR sequence was performed. The detailed parameters of the MRI are presented in Table 1.

**Table 1:** Parameters and sequences of WB-DWI for the evaluation of patients with cancer. WB-DWI: whole-body diffusion-weighted imaging, WB-STIR: whole-body short inversion time inversion recovery, TR: repetition time, TE: echo time.

Sequence	WB-DWI	WB-STIR
Plane	Axial	Axial
Regions	Vertex to mid-thigh	Vertex to mid-thigh
Gap, mm	1	0
Slice thickness (mm)	5	5
TR, ms	9000	4000
TE, ms	58	60
b-value, s/mm^2^	0-800	-
Respiratory motion	Free-breathing	Breath-hold
Total examination time (min)	15-25	10-15

Whole-body raw DWI and STIR images obtained in the axial plane were reformatted. Coronal MPR images were generated on the main console from the axial plane images. Whole-body MPR images were created by stitching the reformatted images. These images were stitched in three sequences to produce whole-body coronal images. DWI with a b-value of 800 s/mm² was converted from grayscale to negative to achieve an appearance similar to PET images.

WB-DWI was evaluated by a radiologist with 14 years of experience using a GE workstation (version 4.7, GE Medical Systems, Milwaukee, Wisconsin). To evaluate metastatic lesions and determine their location, each case was assessed across 18 different regions, including the skeletal system, visceral areas, and lymph nodes. The skeletal system was divided into eight regions: cranium, vertebrae, sternum, ribs, clavicles, scapulae, pelvic bones, and extremities. Visceral areas included the lungs, liver, soft tissue, and brain. Lymph nodes were categorized into cervical, supraclavicular, mediastinal, axillary, abdominal, and inguinal regions. Each of these regions was evaluated for the presence of metastases in every case.

Statistical analysis

The data were analyzed using IBM SPSS Statistics for Windows, Version 25 (Released 2017; IBM Corp., Armonk, New York). For continuous variables, the Mann-Whitney U test was used for comparisons between groups, while the Wilcoxon test was applied for comparisons of dependent groups. The chi-square test was used to evaluate the comparison of discrete variables between groups. A p-value of <.05 was considered statistically significant. Descriptive statistics are presented as numbers and percentage averages. Metastasis detection by WB-DWI was evaluated using k statistics and the chi-square test. The grading of agreement based on the Kappa (κ) value was classified as follows: slight (κ <0.21), fair (κ = 0.21-0.40), moderate (κ = 0.41-0.60), substantial (κ = 0.61-0.80), and almost perfect (κ = 0.81 to <1.00) [3].

## Results

Of the 21 patients included in the study, 12 (57.1%) were men and 9 (42.9%) were women, with a mean age of 58 years.

The primary tumors in each patient had been histopathologically confirmed. The underlying primary tumors in the study population included invasive ductal breast cancer (n = 5), non-Hodgkin lymphoma (n = 2), Hodgkin lymphoma (n = 3), pancreatic serous cystadenocarcinoma (n = 3), colorectal carcinoma (n = 3), endometrial carcinoma (n = 1), ovarian serous cystadenocarcinoma (n = 2), and non-small cell lung carcinoma (n = 2).

Metastatic lesions were evaluated by dividing them into 18 regions: skeletal system, visceral areas, and lymph nodes. A total of 378 regions were examined across all 21 cases. Of the 68 metastases detected by PET/CT, 64 were also identified by WB-DWI (Table 2).

**Table 2:** Distribution and number of metastatic lesions. PET/CT: positron emission tomography/computed tomography, WB-DWI: whole-body diffusion-weighted imaging.

Lesion localization	Number of patients and percentage values	
	PET/CT	WB-DWI
Liver metastasis	3 (4.4%)	2 (3.1%)
Lung metastasis	6 (8.8%)	4 (6.2%)
Adrenal gland metastasis	1 (1.4%)	1 (1.15%)
Soft tissue metastasis	5 (7.3%)	5 (7.8%)
Lymph node localization		
Cervical	5 (7.3%)	5 (7.8%)
Supraclavicular	3 (4.4%)	3 (4.6%)
Mediastinal	6 (8.8%)	6 (9.3%)
Axillary	5 (7.3%)	5 (7.8%)
Abdominal	9 (13.2%)	9 (14%)
Inguinal	3 (4.4%)	3 (4.6%)
Subtotal	31 (45.5%)	31 (48.4%)
Bone metastasis localization		
Cranial bone	2 (2.9%)	2 (3.1%)
Vertebra	5 (7.3%)	4 (6.2%)
Sternum	2 (2.9%)	2 (3.1%)
Costa	5 (7.8%)	5 (7.8%)
Clavicle	2 (2.9%)	2 (3.1%)
Scapula	1 (1.4%)	1 (1.5%)
Pelvic bones	3 (4.4%)	3 (4.6%)
Limb	2 (2.9%)	2 (3.1%)
Subtotal	22 (32.3%)	21 (32.8%)
Total	68	64

In three patients, metastatic liver lesions were detected using PET/CT. Liver metastases were identified in two patients with WB-DWI. In one patient, a lesion located in liver segment 2 was not detected due to the presence of cardiac motion artifacts. The use of WB-DWI imaging for detecting liver metastases was found to be statistically significant and substantial (κ = 0.77; p < .001).

Lung metastases were detected by PET/CT in six patients, and metastases were identified by WB-DWI in four of these patients. WB-DWI imaging for the detection of lung metastases was statistically significant and substantial (κ = 0.74; p < .001). The sensitivity and accuracy of WB-DWI in detecting lung metastases were 66.7% and 92.6%, respectively (Table 2).

All cases of adrenal gland and soft tissue metastases were detected using WB-DWI. The statistical significance of WB-DWI imaging in detecting adrenal gland and soft tissue metastases was confirmed, with perfect agreement (κ = 1; p < .001).

**Figure 1:**
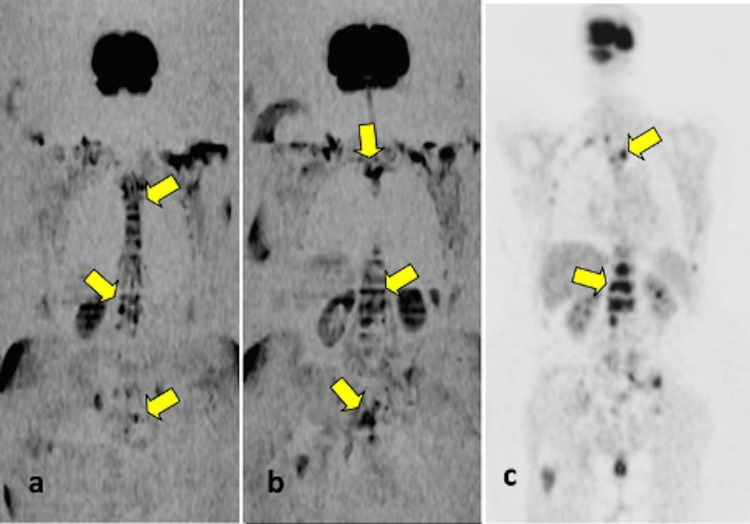
A 62-year-old female patient diagnosed with breast cancer. (a, b) Multiple metastatic signal changes are observed in the vertebrae on coronal inverted whole-body DWI. (c) Coronal PET image shows pathological metabolic activity in the vertebrae. DWI: diffusion-weighted imaging, PET: positron emission tomography.

The PET/CT scans revealed a total of 22 bone metastatic locations. Of these, 21 (95.4%) were detected using WB-DWI (Table 2 and Figure 1).

**Figure 2:**
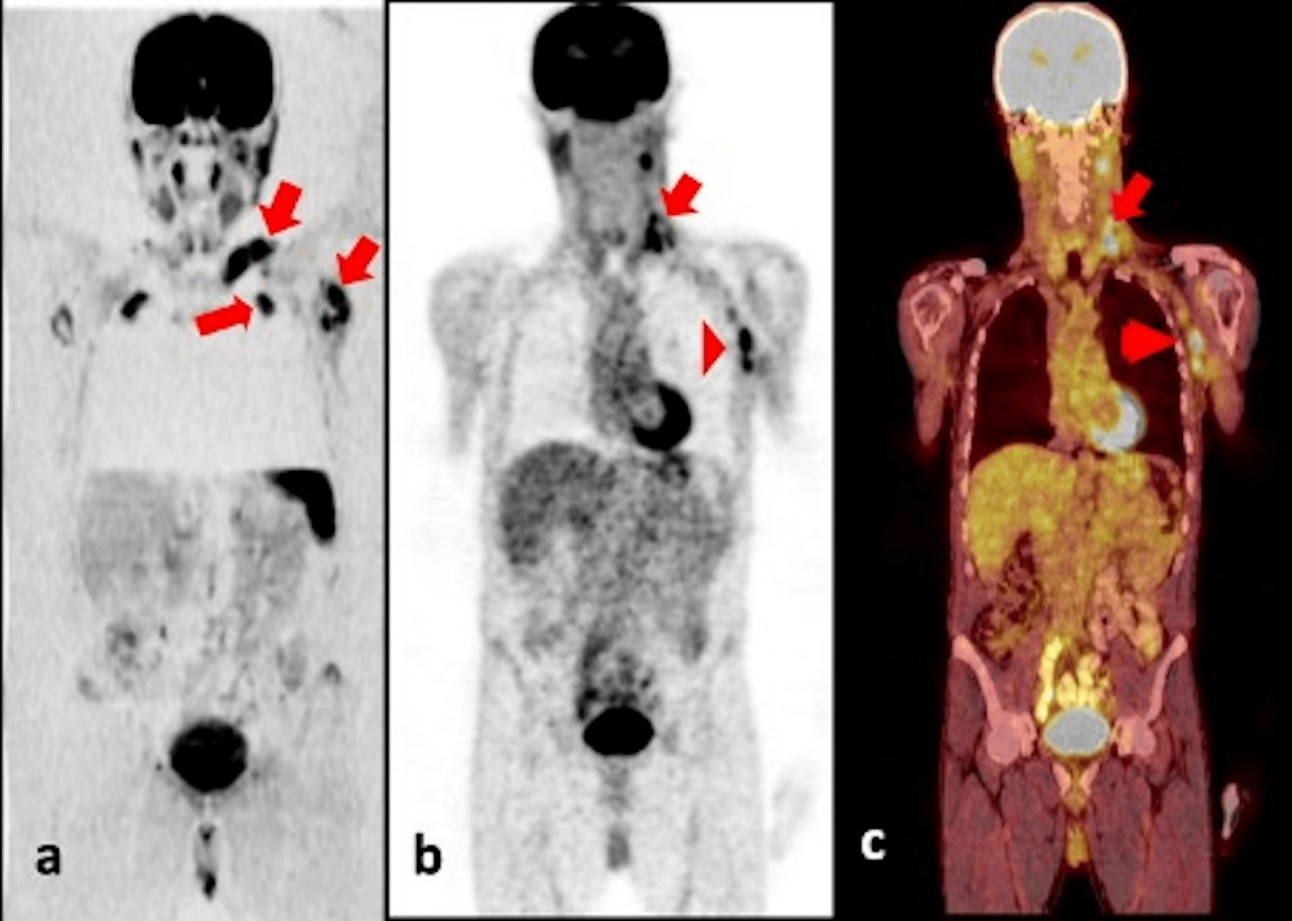
A 62-year-old male patient diagnosed with parotid gland adenocarcinoma. (a) Coronal inverted whole-body DWI examination shows enlarged lymph nodes with pathological signal changes in the left supraclavicular and axillary regions. (b, c) PET and fused PET/CT images reveal increased metabolic activity in the lymph nodes in the left supraclavicular and axillary regions. DWI: diffusion-weighted imaging, PET/CT: positron emission tomography/computed tomography.

**Figure 3:**
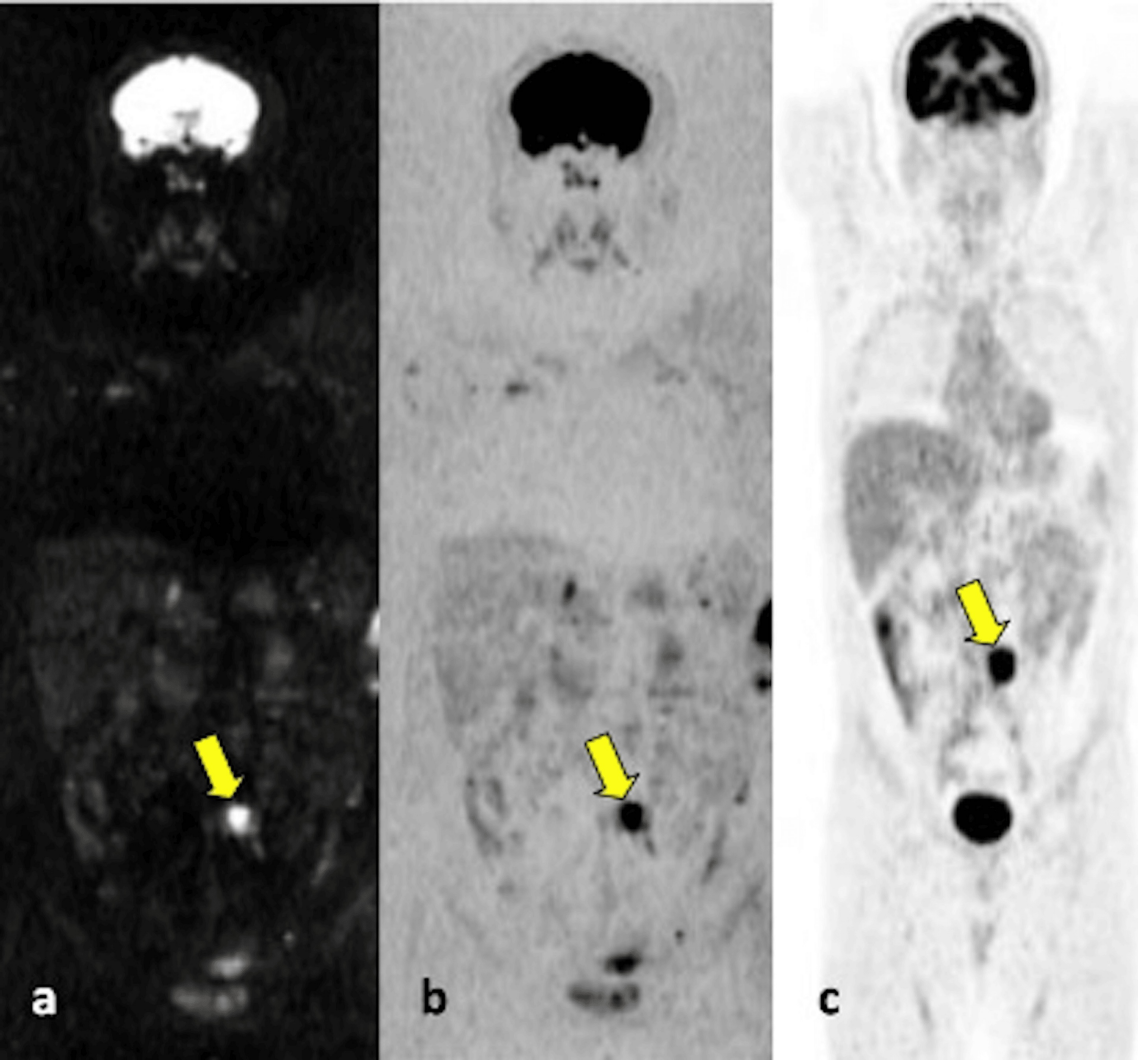
Coronal MPR (a) and inverted (b) whole-body DWI examinations of a 44-year-old male patient diagnosed with seminoma show an increased signal in the left para-aortic enlarged lymph node in the abdomen. Coronal PET (c) images reveal intense 18F-FDG uptake in the enlarged lymph node in the same region. MPR: multiplanar reconstruction, DWI: diffusion-weighted imaging, PET: positron emission tomography, 18F-FDG: fluorine-18 fluorodeoxyglucose.

WB-DWI imaging for the detection of bone metastases was statistically significant and demonstrated almost perfect agreement (κ = 0.90; p < .001). The sensitivity and accuracy of WB-DWI in detecting bone metastases were 85.7% and 96.4%, respectively. In PET/CT scans of 21 patients, 31 metastatic lymph node regions were identified. WB-DWI successfully detected all metastatic lymph nodes (Table 2; shown in Figures 2, 3).

The efficacy of WB-DWI imaging in detecting lymph node metastases was statistically significant, with perfect agreement (κ = 1; p < .001). The sensitivity and accuracy of WB-DWI in detecting lymph node metastases were both 100%.

## Discussion

WB-DWI has proven to be particularly valuable in the evaluation of oncological diseases by leveraging the increased cellularity and decreased extracellular space characteristic of malignancies. This technique enables the detection, characterization, and monitoring of tumors throughout the body and is especially useful in assessing treatment response and detecting metastatic disease, playing a critical role in oncological management [4]. In our study, WB-DWI demonstrated a high level of agreement with PET/CT, detecting 94.1% (94/98) of metastases identified by PET/CT. This finding underscores the potential of WB-DWI as a reliable alternative for metastatic disease evaluation. Consistent with prior research showing the advantages of integrating traditional MR sequences and DWI, we incorporated the WB-STIR sequence into our scanning protocol to enhance anatomical orientation. Our results align with previous studies indicating that WB-MRI, including DWI, is a reliable method for the initial staging of metastatic breast and prostate cancers and can serve as an alternative to PET/CT [5,6].

However, the effectiveness of WB-DWI depends on the histological type, grade, and anatomical location of the tumor. Studies have demonstrated high sensitivity and specificity in small-cell tumors, such as breast cancer, multiple myeloma, lymphoma, neuroendocrine tumors, and small-cell lung cancer [7-10].

Primary tumors and distant metastases often exhibit diffusion restriction, which WB-DWI can detect, making it particularly useful for identifying small lesions [11]. Moreover, WB-DWI facilitates treatment monitoring using established criteria, such as the Response Evaluation Criteria in Solid Tumors (RECIST) version 1.1. It also allows for quantitative assessment of treatment response through changes in apparent diffusion coefficient (ADC) values, which reflect tumor cellularity and the microenvironment [12-14]. These capabilities make WB-DWI a versatile tool for both diagnosis and longitudinal disease management.

PET/CT's reliance on 18F-FDG uptake poses challenges for tumors with low metabolic activity, such as prostate cancer, neuroendocrine tumors, and low-grade lymphomas, where WB-DWI may demonstrate superior sensitivity [15]. Additionally, PET/CT struggles in anatomical regions with high physiological FDG uptake, including the brain, renal collecting system, and bladder, reducing its diagnostic accuracy in these areas [16]. For example, the high FDG uptake in the brain limits PET/CT’s sensitivity for detecting brain metastases, whereas WB-DWI provides superior sensitivity and anatomical resolution for this purpose. These advantages position WB-DWI as a complementary or alternative imaging modality in specific clinical scenarios.

In our study, we observed limitations with WB-DWI, particularly in detecting pulmonary lesions smaller than 6 mm. Lung imaging with WB-DWI is inherently challenging due to susceptibility artifacts, low proton density, and motion artifacts caused by respiration and cardiac pulsation. For example, Regier et al. reported sensitivity rates of 86.4% for nodules measuring 6-9 mm and 97% for nodules ≥10 mm when using DWI [17]. In our cohort, two small lung metastases undetectable by WB-DWI highlight these limitations. However, PET/CT is not without its challenges, including susceptibility to respiratory artifacts that may hinder the accurate fusion of PET and CT images.

Intermittent examinations are often required in the follow-up of oncological patients, increasing radiation exposure. This concern can be mitigated with the widespread clinical application of WB-DWI. Additionally, unlike PET/CT, DWI can be used safely in pregnant and pediatric patients. Since WB-DWI does not involve ionizing radiation, it is especially advantageous for whole-body scanning in these populations [18,19].

Although our results suggest that WB-DWI has comparable sensitivity to PET/CT for screening metastases, several practical challenges may limit its clinical application, including longer acquisition times compared to PET/CT, scheduling difficulties due to limited scanner availability, and patient-related factors such as claustrophobia. Several strategies can address these issues. First, advances in MRI technology, such as faster imaging protocols and the use of compressed sensing, could reduce acquisition times. Second, patient-centered approaches, such as pre-scan counseling, sedation when appropriate, and the use of open or wider-bore MRI systems, could improve patient comfort and mitigate issues like claustrophobia.

Limitations

Although our study provides promising results for WB-DWI in detecting metastatic lesions, it has some limitations. First, the sample size of our study group was limited, which could have an impact on how broadly applicable our findings are. Additionally, the diversity of malignancies included in the study was low. Another limitation of WB-DWI is the potential for non-malignant diffusion-restricting lesions, such as infections, abscesses, or inflammatory processes, to mimic malignant lesions. This overlap can lead to diagnostic uncertainty or false-positive findings. In this study, we minimized this risk by correlating imaging findings with clinical and laboratory data, and, when available, histopathological confirmation. However, this approach does not completely eliminate the possibility of misclassification. Larger patient populations in future research are required to validate and further clarify the clinical utility of WB-DWI in evaluating metastatic cancer.

## Conclusions

WB-DWI demonstrated a high level of concordance with PET/CT in detecting metastases, underscoring its potential as a radiation-free alternative for comprehensive oncological imaging. While certain limitations and practical challenges persist, ongoing advancements in MRI technology and patient-centered imaging strategies may further enhance the utility of WB-DWI. Future research should aim to address these limitations, validate our findings in larger cohorts, and refine the clinical applications of WB-DWI to maximize its diagnostic and therapeutic impact.
